# Histone deacetylases as new therapy targets for platinum-resistant epithelial ovarian cancer

**DOI:** 10.1007/s00432-015-2064-5

**Published:** 2015-11-11

**Authors:** Dmitri Pchejetski, Albandri Alfraidi, Keith Sacco, Heba Alshaker, Aun Muhammad, Leonardo Monzon

**Affiliations:** School of Medicine, University of East Anglia, Bob Champion Research and Education Bldg 2.53, Norwich, UK; Department of Surgery and Cancer, Hammersmith Hospital, Imperial College London, 1st Floor ICTEM, Du Cane Road, London, W12 0NN UK; University of Malta Medical School, Malta, Malta; Faculty of Pharmacy and Medical Sciences, University of Petra, Amman, Jordan; Department of Imaging, St Mary’s Hospital, London, UK

**Keywords:** HDACS, Platinum resistance, Ovarian cancer, Platinum, Chemotherapy, Molecular targeting

## Abstract

**Introduction:**

In developed countries, ovarian cancer is the fourth most common cancer in women. Due to the non-specific symptomatology associated with the disease many patients with ovarian cancer are diagnosed late, which leads to significantly poorer prognosis. Apart from surgery and radiotherapy, a substantial number of ovarian cancer patients will undergo chemotherapy and platinum based agents are the mainstream first-line therapy for this disease. Despite the initial efficacy of these therapies, many women relapse; therefore, strategies for second-line therapies are required. Regulation of DNA transcription is crucial for tumour progression, metastasis and chemoresistance which offers potential for novel drug targets.

**Methods:**

We have reviewed the existing literature on the role of histone deacetylases, nuclear enzymes regulating gene transcription.

**Results and conclusion:**

Analysis of available data suggests that a signifant proportion of drug resistance stems from abberant gene expression, therefore HDAC inhibitors are amongst the most promising therapeutic targets for cancer treatment. Together with genetic testing, they may have a potential to serve as base for patient-adapted therapies.

## Overview of ovarian cancer

Ovarian cancer is the leading cause of death arising from gynaecological malignancies and the fourth most common form of cancer in women in developed countries, after breast, lung, and colorectal cancer (Permuth-Wey and Sellers 2009; Gayther and Pharoah 2010). Around 204,000 new cases of ovarian cancer are diagnosed worldwide yearly with the highest incidence being in the USA and Northern Europe (Rauh-Hain et al. 2011). The disease is prevalent in older, post-menopausal women and over 80 % of cases are diagnosed in women over 50 years of age (Herzog and Pothuri 2006). The main risk factors associated with ovarian cancer are family history, infertility, and increasing age (Tortolero-Luna and Mitchell 1995; Cetin et al. 2008). The estimated risk of developing ovarian cancer in monozygotic twin of an affected patient is twice the non-twin sibling risk, implies that familial risk of ovarian cancer may be more related to genetic factors than to shared environmental effects (Gayther et al. 2007). Further, positive correlations were observed with dietary consumption of fats and proteins (Bosetti et al. 2001).

Initial symptoms of ovarian cancer prior to diagnosis are non-specific and include vaginal bleeding, gastrointestinal discomfort, and urinary tract symptoms (Friedlander 1998). Further, over 95 % of epithelial ovarian cancer patients experience abdominal complaints for many months prior to their diagnosis (Goff et al. 2007; Lowe et al. 2009). The initial diagnostic workup includes a pelvic examination, ultrasound examination, computed tomography scans, and a blood test for cancer antigen 125 (CA 125), yet none of these techniques can be reliably used for early detection. CA-125 is a tumour marker produced by ovarian cancer cells that is detectable in the serum of >80 % of women with ovarian carcinomas (Klug et al. 1984). However, it is raised in a number of benign ovarian disorders as well as pregnancy, rendering it mostly useful for monitoring treatment response or disease recurrence (Tuxen et al. 1995; Marsden et al. 2000; Meyer and Rustin 2000). Ovarian cancer has four surgical stages according to the International Federation of Gynecology and Obstetrics (FIGO) staging system (Table 1), which outlines patient prognosis and treatment (Bast et al. 2009).

**Table 1:** Ovarian cancer FIGO classification

Stage	Description
I	Ovarian cancer limited to one or both ovaries
II	Cancer spread outside the ovary or ovaries, but it is inside the pelvis
III	Cancer has grown outside the pelvis into the abdominal cavity or involvement of upper abdominal, inguinal or retroperitoneal lymph nodes
IV	Cancer has spread into other body organs such as the liver or outside the peritoneal cavity

According to the International Federation of Gynecology and Obstetrics (FIGO) staging system, ovarian cancer is categorised into four surgical stages (described above). These stages outline treatment options, and a higher stage is associated with a worse prognosis. Adapted from (Benedet et al. 2000)

Ovarian cancer is broadly classified into three cellular and histological subtypes. The cellular classification comprises epithelial tumours, germ cell (ova) tumours and the sex cord (stromal) tumours (Kurian et al. 2005). Histologically, each ovary is covered with germinal epithelium cells beneath which are two regions; an outer cortex and inner medulla responsible for oogenesis and neurovascular support, respectively (Naora and Montell 2005). Every histological subtype of ovarian cancer has different molecular genetic changes underlying its progression. Epithelial ovarian cancer is the most common type of malignant ovarian tumour, occurring in 90 % of cases. Epithelial ovarian cancer is morphologically divided into four distinct subtypes; serous is the most malignant tumour of the epithelial ovarian cancer followed by endometrioid, clear cell and mucinous (Bowtell 2010). This review presents an overview of epithelial ovarian cancer, drug resistance and relapse and a particular focus on the role of histone deacetylase inhibitors as novel drugs in the field.

## Biology of epithelial ovarian cancer

Most ovarian cancers arise from the surface epithelium (Piek et al. 2004) yet the biological mechanism behind this transformation is not precisely understood. The theory of incessant ovulation attributes repeated disruption of the ovarian epithelium that leads to malignant transformation of the epithelial cells (Fathalla 1971; Booth et al. 1989). Factors that suppress incessant ovulation include pregnancy and use of contraceptive that are associated with reduced risk of developing ovarian cancer. Hormones including gonadotropins and sex steroids are thought to influence tumorigenesis. Excessive gonadotropin secretion during menopause may increase the risk of malignant alterations in the ovaries by stimulating the growth of ovarian epithelial cells (Risch 1998). It is debatable whether high androgen levels may increase risk of ovarian cancer. While it has been shown that 90 % of ovarian cancer tissue were positive for the androgen receptor (Wang and Chang 2004) a case–controlled study showed no association between serum levels of androgens and ovarian cancer risk (Rinaldi et al. 2007).

Ovarian cancer development involves the accumulation of several genetic alterations that, respectively, activate or silence oncogenes and tumour suppressor genes (Gayther et al. 1997; Schuijer and Berns 2003). Inherited ovarian cancer comprises 5–15 % of ovarian cancer (Boyd 1998). It is associated with improved overall survival and better clinical outcome than sporadic cancer. This may be due to an earlier diagnosis and subsequent earlier treatment. The BRCA1 and BRCA2 tumour suppressor genes are the most important predisposition genes for ovarian cancer and account for about 90 % of ovarian cancers in the hereditary breast and ovarian cancer syndrome (Gayther et al. 1997; Stratton et al. 1997). BRCA1 and BRCA2 are key proteins that mediate DNA double-strand break repair (D’Andrea and Grompe 2003). Germ line mutations in the DNA mismatch repair (MMR) genes (hMLH1, hMSH2, hMSH6, PMS1, PMS2) which are commonly associated with non-polyposis colorectal cancer (HNPCC) are also associated with ovarian cancer. Ovarian cancer risk in HNPCC families is more than 12 % (Prat et al. 2005). The genes associated with familial ovarian cancer are also involved in disease pathogenesis of the more prevalent sporadic ovarian cancer.

A mutation of the p53 gene is the most common genetic alteration in epithelial ovarian cancers. This tumour suppressor gene encodes a transcription factor that has a role in regulating cell cycle progression, DNA repair, and cell death. The majority of early and advanced stage serous carcinomas have mutant p53 (Havrilesky et al. 2003; Leitao et al. 2004; Palmer et al. 2008). PTEN tumour suppressor (phosphatase and tensin homolog) is one of the mutated tumour suppressor genes in ovarian cancer and has a role in cell apoptosis, proliferation and migration (Kolasa et al. 2006; Goff et al. 2007). The RAS family consists of three functional genes, Hras, K-ras, and N-ras that are involved in signal transduction. The K-ras mutation is a common feature in ovarian mucinous carcinomas (Auner et al. 2009). The combination of activating mutations in the oncogenic K-ras gene and PTEN deletion plays a role in the development of endometrioid epithelial ovarian cancer (Dinulescu et al. 2005). The c-MYC proto-oncogene, which participates in the regulation of cellular proliferation, apoptosis and cell differentiation, has also been shown to be overexpressed in endometrioid and clear cell carcinomas (Plisiecka-Halasa et al. 2003). Genes related to the WNT/b-catenin and cadherin signalling pathways were overexpressed in high-grade serous tumours, including N-cadherin and P-cadherin (Tothill et al. 2008). Such data show that each subtype of ovarian cancer has distinct gene mutations. Gene expression analyses can distinguish histological subtypes based on their global gene expression profiles (Cho and Shih Ie 2009; Gomez-Raposo et al. 2010).

## Treatment of ovarian cancer

Treatment of ovarian cancer broadly encompasses surgery, radiotherapy and chemotherapy. Treatment depends upon various parameters such as stage, the histopathologic type, patient’s age, patient’s desire to have children and overall health status (Cannistra 2004; Marcus et al. 2014). Surgery prior to chemotherapy depends on the size of the tumour and the patient condition. Platinum-based chemotherapy has been used to reduce the size of the tumour before surgery. While neoadjuvant chemotherapy makes surgical operation easier, surgical reduction of tumour bulk prior to chemotherapy might reduce the chance of developing chemo-resistance (Morrison et al. 2007). Radiotherapy can be used to treat residual cancer after cytoreductive surgery and chemotherapy (Bese et al. 2009). The size and the site of residual disease and tumour grade determine the successful outcome (Dembo 1983; Einhorn et al. 2003). Radiotherapy could enhance survival rate when combined with surgery and chemotherapy (Einhorn et al. 1999).

Neoadjuvant or adjuvant chemotherapy is administered to most ovarian cancer patients. Chemotherapy drugs are categorised into several groups based on their chemical structure and mechanism of action (Table 2). Platinum-based agents are the first-line choice drugs. The standard treatment for ovarian cancer patients is combination therapy using carboplatin and paclitaxel due to its effectiveness and favourable toxicity profile (Ozols et al. 2003). Chemotherapy can be administered via the oral, intravenous or intraperitoneal (IP) with the latter showing an improved progression-free survival (Markman 2001; Armstrong and Brady 2006). The side effects of IP therapy can be severe such as the toxicity, catheter-related problems, abdominal pain, bloating, fatigue, infection and gastrointestinal disturbance (Walker et al. 2006; Fung-Kee-Fung et al. 2007).

**Table 2:** Cytotoxic chemotherapy groups active in ovarian cancer and their mechanism of action (Dunton 1997; Piccart et al. 2001)

Group	Action and example
Anti-metabolite drugs	These interfere with DNA and RNA synthesis e.g., 5-fluorouracil
Anti-tumour antibiotics including topoisomerase II	These inhibit DNA and RNA synthesis leading to cell death such as doxorubicin
Plant-based compounds	These are natural products of plant alkaloids that can inhibit mitosis or enzymes, thereby preventing protein synthesis necessary for cell like etoposide and taxanes
Alkylating agents	These interfere with DNA thus prevent the cancer cells growth
Platinum-based agents	are the most effective chemotherapy for ovarian cancer on whole, showing high response rates and long survival for advanced ovarian cancer patients such as cisplatin and carboplatin

Platinum-based therapy is very effective in the treatment of ovarian cancer. However, patients frequently develop platinum resistance. Resistance can be either acquired, when the patients respond to the chemotherapy but then develop resistance afterwards or intrinsic, when the patients are unaffected by platinum treatment at first exposure (refractory). Drug resistance is a multi-factorial phenomenon involving various mechanisms. These can be due to decreased platinum uptake or increased platinum-DNA adduct repair and damage tolerance. Further, cellular glutathione or metallothionein cause increased efflux of cisplatin. Alternatively, there could be alterations in the signalling pathways affecting apoptosis (Kartalou and Essigmann 2001; Wernyj and Morin 2004; Ohmichi et al. 2005).

At present there are a number of drugs that are emerging as potential therapeutics in ovarian cancer. Sorafenib is a receptor tyrosine kinase inhibitor. It inhibits protein kinases such as vascular endothelial growth factor receptor (VEGFR) and platelet-derived growth factor receptor (PDGFR) together with rat sarcoma proto-oncogene (RAS), rat fibrosarcoma protein kinase (RAF) and mitogen-activated protein kinase (MAPK) (Matei et al. 2011; Zhang et al. 2013). The expression of histone deacetylases (HDACs) is increased in cells undergoing epithelial-mesenchymal transformation; however, the expression of HDACs was not increased in the presences of sorafenib. This suggests that sorafenib may block the expression of HDACs (Zhang et al. 2013). Some ovarian cancer patients on sorafenib experienced stable disease for up to 1 year in a phase II trial (Strumberg et al. 2005). In a phase III trial, patients with ERK and b-RAF positive tumours had neoadjuvant sorafenib at a dose of 800 mg daily. 24 % had a 6 month progression-free survival (Matei et al. 2011). So far sorafenib trials have targeted patients with advance ovarian cancer and trials were not well-standardised; thus, its role in ovarian cancer has yet to be defined (Smolle et al. 2014).

Angiogenesis is a critical step in ovarian cancer metastasis; thus, anti-angiogenic drugs that target VEGF and PDGF have a role to play in ovarian cancer treatment (Conteduca et al. 2014). Bevacizumab, an anti-VEGF monoclonal antibody, has been tried as a single agent and in combination with platinum-based chemotherapy in recurrent or metastatic epithelial ovarian cancer (Burger et al. 2007; Kudoh et al. 2011; Sorbe et al. 2012). The treatment regimens are generally well tolerated and emerging results suggest improved patient efficacy (McGonigle et al. 2011; Gavalas et al. 2013). A phase III trial showed that bevacizumab improved chemotherapy efficacy and progression-free survival in initial disease management (Burger et al. 2011; Perren et al. 2011). Similar outcomes were obtained in platinum-sensitive ovarian cancer (Aghajanian et al. 2012). Aflibercept is a fusion protein of VEGFR1 and VEGFR2 attached to the Fc portion of human IgG. The safety profile of the drug in combination with docetaxel is established. Preliminary results are promising and warrant further investigation (Coleman et al. 2011). A number of other anti-angiogenic drugs have undergone pre-clinical studies (Wedge et al. 2005; Bauerschlag et al. 2010; Karlan et al. 2012). Despite their efficacy, patients can develop drug resistance that may be due to cancer cell utilisation of alternative angiogenic pathways and recruitment of vascular progenitor cells (Conteduca et al. 2014).

One mechanism that underlies ovarian tumour progression, metastasis and chemoresistance is transcriptional regulation. TATA-box binding protein-associated factors (TAFs) regulate dedifferentiated states in ovarian cancer (Ribeiro et al. 2014). Understanding the nuclear mechanisms associated with treatment failure in ovarian cancer would uncover novel pharmacologic targets to treat patients.

## Histone deacetylase (HDACS)

### The biological function of histone deacetylases (HDACs)

**Fig. 1:**
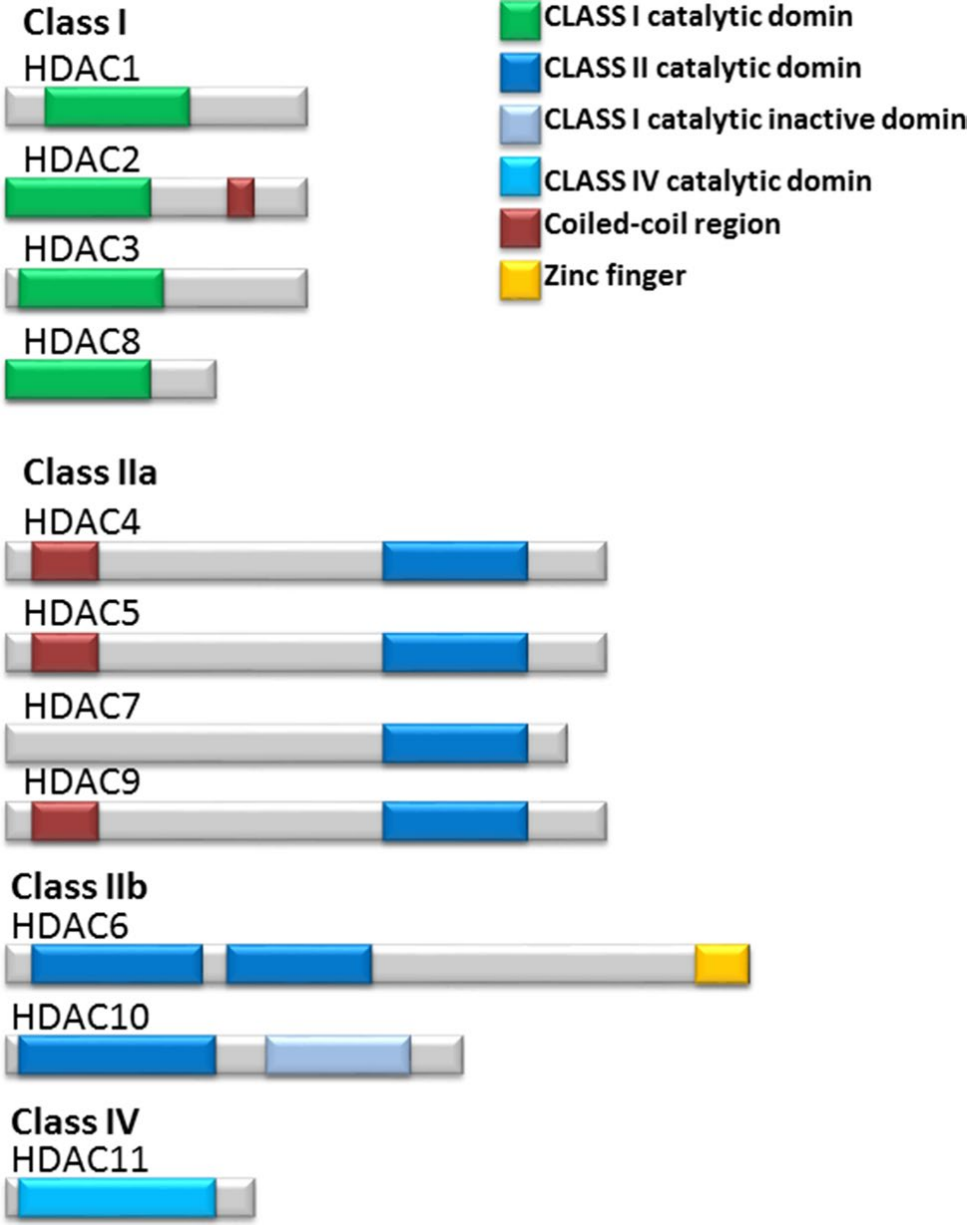
Histone acetylation/deacetylation regulates transcription. The balance between the acetylated and deacetylated states of histones is mediated by two different sets of enzymes: histone acetyltransferases (HATs) and histone deacetylases (HDACs). Acetylation of the histone tails is associated with an open chromatin structure and active transcription. Conversely, removing the acetyl groups on the histone tails by HDAC enzyme activity is associated with condensed chromatin structure and transcriptional repression. Adapted from Marks et al. (2001)

Transcription in eukaryotic cells is influenced by the manner in which DNA is packaged. DNA is packaged into chromatin, a highly organised protein DNA complex. The basic units of the chromatin are nucleosomes. It is comprised of four histones; two H2A, H2B dimmers and H3, H4 tetramer. The presence of acetylated lysine residues in histone tails is associated with a more relaxed chromatin state and gene-transcription activation, while the deacetylation of lysine residues is associated with a more condensed chromatin state and transcriptional gene silencing (Fig. 1) (Strahl and Allis 2000). Thus, the balance between histone acetyltransferase (HAT) and histone deacetylase (HDAC) activities play a crucial role in chromatin remodelling and in the regulation of gene transcription. HDACs family contained 18 known enzymes.

**Fig. 2:**
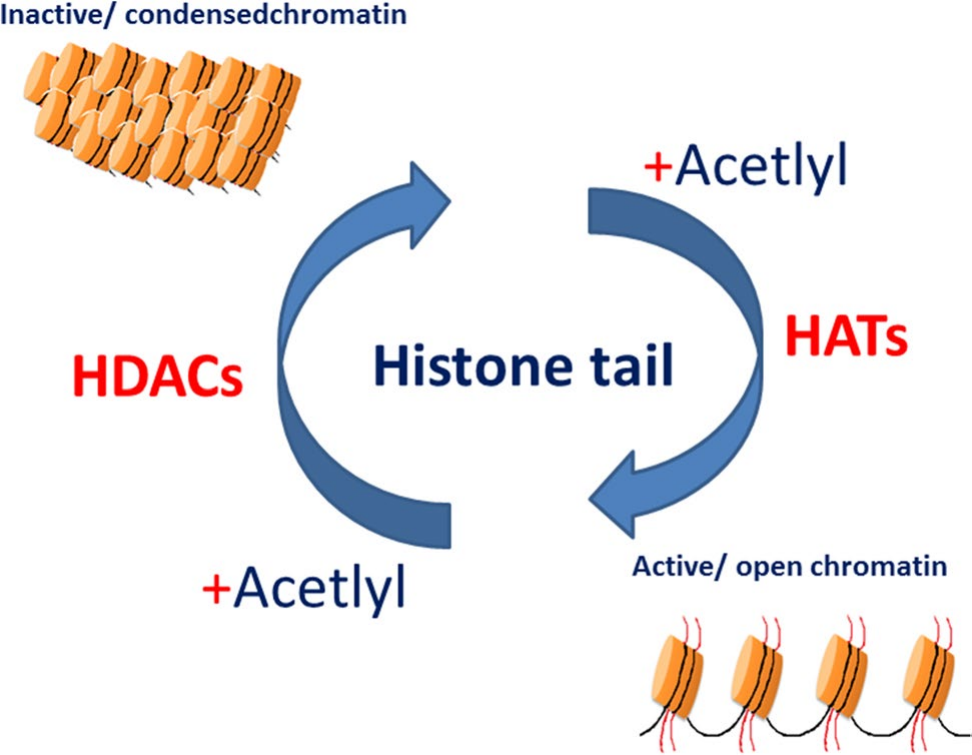
Schematic representation of class I; HDAC1, 2, 3 and 8, class IIa; HDAC4, 5, 7 and 9, class IIb; HDAC6 and 10 and class IV; HDAC11. HDAC enzymes with the structural and the functional domains included the capacity of the structurally diverse HDAC inhibitors to inhibit the activity of each HDAC classes or specific isoform HDACs are shown. Adapted from Bolden et al. (2006)

These HDAC enzymes divided into four classes based on sequence similarity to the yeast (Fig. 2). The class I, II, and IV HDACs are zinc-dependent and class III HDACs (SIRT1–SIRT7) are NAD+ dependent in their enzymatic activity class I HDACs include HDAC1, 2, 3, and 8. They are 400 residues in length and are considered as nuclear proteins; however, HDAC3 has the ability to shuttle between the nucleus and the cytoplasm. Class II HDACs include HDAC4, 5, 6, 7, 9, and 10. These enzymes have the ability to shuttle between the nucleus and cytoplasm. In addition, class II HDACs enzymes are grouped into class IIa (HDAC4, 5, 7, and 9) and IIb (HDAC6 and 10) because class IIa enzymes have N-terminal extension of 600 residues that are thought to have distinctive function, but class IIb enzymes have two catalytic domains. Class IV, share the common properties between class I and class II HDACs and includes HDAC11 as reviewed in (Verdin et al. 2003). Class I enzymes are ubiquitously expressed in humans, whereas class II enzymes show tissue specific expression. Three of the class IIa HDACs, HDAC4, 5 and 9, show highest expression in heart, skeletal muscle and brain. The highest expression of HDAC7 is in heart and lung tissues. HDAC6 is predominantly expressed in testis, while HDAC10 is expressed in liver, spleen and kidney (Verdin et al. 2003). The HDACs are not redundant in their biological activity. Knockout mouse experiments demonstrated a different function for each HDAC. The class I genes HDAC1 or HDAC2 knockout in mice resulted in embryonic or perinatal lethality, respectively. However, class II HDAC knockout mice were viable and fertile, but all display developmental abnormalities. HDAC7 null mouse embryos have defects in blood vessel development and integrity. HDAC9 null mice display defects in cardiac muscle development, reviewed in (Lane and Chabner 2009).

Both class I and class II HDACs act on histone substrates and on non-histone proteins which play important roles in the regulation of various cellular processes. HDAC1 has been reported to affect the deacetylation of the transcription factor p53, while HDAC6 deacetylate α-tubulin and heat-shock protein 90 (HSP90) (Bolden et al. 2006). HDAC enzymes exist in multi-protein complexes and recruited to certain promoters by interaction with specific transcription factors although these enzymes are unable to bind directly to DNA. For example, HDAC1 and HDAC2 are thought to be found together in multi-protein complexes, and a number of transcription factors target these two enzymes to specific promoters to suppress transcription (MacDonald and Roskams 2008). Amongst all HDAC enzymes, only HDAC3 has the ability to interact with class I and II HDACs. HDAC3 interacts with HDAC4, SMRT (silencing mediator for retinoid and thyroid receptors) and N-CoR (nuclear receptor co-repressor) complexes to repress transcription. The suppression of HDAC4 binding to SMRT and N-CoR and to HDAC3 lead to a loss of enzymatic activity related to HDAC4. It has been reported that class II HDACs require an enzymatically active SMRT/N-CoR-HDAC3 complex to regulate transcription (Grozinger et al. 1999; Fischle et al. 2002) with the exception of HDAC6 which contains an internal dimer including two functional catalytic domains and thus does not require another HDAC enzyme for its activity (Grozinger et al. 1999). HDAC6 has been shown to specifically deacetylate α-tubulin at lysine 40. Therefore, this lysine has been used as a marker of microtubule stability (Piperno et al. 1987). Sirt1, a class III HDAC, has been reported to deacetylate the p53 gene and to inhibit the transcriptional activity and apoptosis mediated by this gene (Cheng et al. 2003). Class II HDACs can deacetylate the four core histones and act like transcriptional corepressors. These enzymes either act through transcriptional corepressors or bind directly to sequence-specific transcription factors, such as myocyte enhancer factor-2 (MEF2). The MEF2 family of transcription factors is one of the important targets of class IIa HDACs. MEF2, major transcriptional activators for the expression of muscle-specific genes, also regulate other cellular programs, including neuronal survival, T cell apoptosis, and growth factor responsiveness. When class IIa HDACs bind to MEF2, it change MEF2 from being transcriptional activators into repressors. The MEF2-binding site is conserved amongst all four class IIa HDACs enzymes (Gregoire and Yang 2005).

The homodimeric and heterodimeric interactions of HDAC might indicate that these interactions are important for the regulation of HDAC activity. Histones not only undergo acetylation and deacetylation but undergo different post-translational modifications such as; phosphorylation, methylation, ubiquitination and sumoylation which are thought to influence the chromatin structure, DNA accessibility and gene expression regulation (Struhl 1998).

### The role of HDACs in cancer

The role of individual histone deacetylases (HDACs) in the regulation of cancer cell proliferation was investigated using siRNA-mediated knockdown (HDAC1, HDAC2, HDAC3, HDAC4 and HDAC7) in HeLa cells. Proliferation analysis showed that knockdown of HDAC1 and HDAC3 resulted in slower cell growth compared with HDAC4 and HDAC7 and that only HDAC1 and HDAC3 are important in cellular proliferation (Glaser et al. 2003). Another study showed that siRNA knockdown of HDAC2 can arrest the cell cycle either at the G(1) phase or during G(2)/M transition, resulting in the loss of mitotic cells and cell growth inhibition. In contrast, HDAC2 knockdown showed no effect on cell proliferation (Senese et al. 2007). Furthermore, HDACs 1 and 2 share a high degree of homology and coexist within the same protein complexes. In spite of their close association, each possesses unique functions. High expression of HDAC2 has been seen in colorectal cancer at polyp stage (more than HDAC1 expression), suggesting that HDAC2 might be involved in the early events of cancer development (Huang et al. 2005). The up-regulation of HDAC3 protein expression has been reported in human colon tumours and knocking down of HDAC3 expression by RNA interference in colon cancer cell lines resulted in growth inhibition, a decrease in cell survival and increased apoptosis. Similar effects were observed for HDAC2 and, to a lesser extent, for HDAC1 (Wilson et al. 2006). HDAC8 has been implicated in smooth muscle cell contractility (Waltregny et al. 2005) although its knockdown by RNA interference inhibits growth of human lung, colon, and cervical cancer cell lines (Vannini et al. 2004). This shows that class I HDACs are essential for cell cycle regulation, proliferation and survival with the exception of HDAC2 which is involved in the regulation of apoptosis in tumour cells. Altered expression of certain HDACs has been reported in tumours compared with normal cells. Increased expression of HDAC1 has been found in gastric cancers, oesophageal squamous cell carcinoma, hormone-refractory prostate cancer, and colon cancer (Choi et al. 2001; Wilson et al. 2006). HDAC2 expression is increased in human colon cancer (Zhu et al. 2004). HDAC3 expression is increased in human tumour samples of colon cancer compared with normal tissue (Shebzukhov et al. 2005). HDAC5 is down-regulated in colon cancer (Scanlan et al. 2002). Class I HDACs have been shown to be expressed at significantly higher levels in ovarian cancers in comparison with normal ovarian tissues, with no significant difference in Class II HDAC expression between the two groups (Khabele et al. 2007). Furthermore, the high expression of class 1 HDACs (HDAC1, 2, and 3) has been associated with poor prognosis in endometrioid subtypes of ovarian and endometrial carcinomas (Weichert et al. 2008). In addition, the expression levels were considerably different in specific tumour histological subtypes with mucinous carcinomas showing the highest positivity rates (71 %); followed by high-grade serous (64 %), clear cell (54 %) and endometrioid subtypes (36 %). HDAC class I expression was generally higher in strongly proliferating tumours (Khabele et al. 2007; Weichert et al. 2008). A recent study using 115 ovarian tumour tissues confirmed that expression of nuclear HDAC1, HDAC2 and HDAC3 proteins increased stepwise in benign, borderline and malignant tumours. Analysis of the effect of specific isoforms of HDACs using siRNA against HDAC1, HDAC2 and HDAC3 on the ovarian carcinoma cell lines, SKOV3, OVCAR3, IGROV-1, ES-2, TOV112D, A2780 and A2780/CDDP showed that knockdown of HDAC1 considerably reduced the proliferation of ovarian carcinoma cells, while knockdown of HDAC3 reduced cell migration (Hayashi et al. 2010). This indicates that HDAC enzymes especially class I have important roles in ovarian carcinogenesis which provides a rationale for targeted inhibition of HDAC enzyme in the treatment of ovarian cancer.

### Therapeutic implications of HDAC inhibitors

HDAC inhibitors are amongst the most promising therapeutic targets for cancer treatment due to their critical role in the regulation of transcription of key genes controlling important cellular functions such as cell proliferation, cell cycle regulation and apoptosis (Spiegel et al. 2012). The balance between histone acetylation and deacetylation is required for active gene expression (Secrist et al. 2003). The catalytic domain of HDAC is closely conserved from bacteria to humans, and the knowledge of its crystal structure has helped in the development of potent HDAC inhibitors (HDACi) based on the molecular mode of action of HDAC. HDAC inhibitor usually consist of a metal-binding moiety that binds the catalytic metal atom within the HDAC active site and a capping group that interacts with the residues at the entrance of the active site (Marks et al. 2001). A linker correctly positions the metal-binding moiety and capping group for interactions in the active site. Crystallographic studies have revealed that SAHA (suberoylanilide hydroxamic acid or vorinostat) inhibits HDAC activity by binding to the active site of the enzyme (Finnin et al. 1999; Marks et al. 2001). A number of natural and synthetic compounds have been shown to be able to inhibit the activity of class I, II, and IV HDACs. HDACi can be divided into several classes: hydroxamic acids, e.g., trichostatin A (TSA) and suberoyl anilide bishydroxamic acid (SAHA), short-chain fatty acids, e.g., sodium butyrate (NaB), cyclic tetrapeptides (e.g., traposin), and benzamides (e.g., MS-275) (Villar-Garea and Esteller 2004). The efficacy of HDAC inhibitors as anticancer agents has been demonstrated in a wide range of haematological and solid tumour cell lines and in experimental animal models (Saito et al. 1999; Drummond et al. 2005).

HDAC inhibitors exhibit the anticancer effects through regulation of gene expression by histone acetylation and through increasing acetylation of other cellular proteins. The molecular pathways that trigger cell death by HDAC inhibitor in ovarian cancer are not fully understood. But there are a number of genes identified to be either up or down-regulated following HDACi treatment. These genes involved in a variety of cellular processes and networks such as those involving the proapoptotic proteins; Fas, Fas ligand, Bak, Bax, Bim, BLxl, Bcl2, Caspase 3, and the cell cycle regulation proteins such as p21 and p27 (Drummond et al. 2005). HDACi treatment has been reported to activate caspase-9, caspase-3, and caspase-8 and inhibit anti-apoptotic Bcl-2 family members (Bcl-2 and Bcl-XL) in breast, colon, hematopoietic, lung, melanoma, ovarian, prostatic, renal, and stomach cancer cell lines using a novel hydroxamate-based HDACI, CG0006 (Hwang et al. 2009). BcL2 proteins control the intrinsic pathway of apoptosis at the mitochondria by preventing the release of the cytochrome c. Therefore, the inhibition of Bcl-2 and Bcl-XL by HDACi treatment promotes the release of cytochrome c followed by caspase-9 and caspase-3 activation leading to apoptosis. As discussed previously, the expression of Bcl2 has been shown to protect ovarian cancer cells from the toxic effect of cisplatin (Hwang et al. 2009). Thus, HDACi could play role in cisplatin resistance in ovarian cancer by modifying the expression of the genes could confer resistance to cisplatin. It has been shown that HDACi treatment induced the accumulation of both proteins p21 and p27, which express in low level in untreated ovarian cancer cell lines and have important role in cell cycle regulation (Hwang et al. 2009). Moreover, specific knockdown by siRNA against HDAC4 in ovarian carcinoma IGROV-1 induced the expression of p21 (Mottet et al. 2009). The up-regulated p21 and p27 bind to cyclin-CDK complexes to inhibit their catalytic activity and induce cell cycle arrest in cancer cells. The study showed that over-expression of P21 and p27 correlates with increased apoptosis, demonstrating that p21 and p27 levels might be essential for HDACi—induced apoptosis (Abukhdeir and Park 2008). This is just one of the possible pathways that HDACi utilise to arrest cancer cell growth. It has also been demonstrated that histone deacetylase inhibitors exhibit anti-cancer activity and induces apoptosis in ovarian cancer cells via up-regulation of E-cadherin expression and accumulation of acetylated histones H3 and H4. The study examined the effect of HDACi (SAHA, VPA, TSA, and NaB) using human ovarian cancer cell lines (SKOV-3, OVCAR-3, TOV-21G, OV-90, TOV-112D, OVCA420, OVCA429, OVCA432, and OVCA433) (Takai et al. 2004).

Hayashi et al. (2010) has reported that knockdown of HDAC3 by siRNA reduced the cell migration with elevated E-cadherin expression in ovarian cancer cells. E-cadherin is one of the important molecules for adhesion between adjacent epithelial cells. When the ovarian epithelial cell undergoes transformation, the cell can detach from the original basement membrane and metastasize throughout the peritoneal cavity. E-cadherin is membrane glycoproteins that mediate adhesion between cells which is an important event in metastasis. Loss of E-cadherin has been associated with ovarian cancer metastasis (Sawada et al. 2008). E-cadherin might have tumour suppressor function in ovarian carcinoma because it has been reported to be silenced by DNA hypermethylation in human breast and prostate carcinomas (Graff et al. 1995). The up-regulated E-cadherin after HDACi treatment in ovarian carcinoma cells indicate that this gene also involved in HDACi meditating cell death in ovarian cancer (Takai and Narahara 2007).

The generation of reactive oxygen species (ROS) is another phenomenon observed in HDAC inhibitor-treated cells. It has been reported that HDAC inhibitors; TSA, MS-275, FK228 and HC toxin promote the induction of DNA damage by a variety of anticancer agents; doxorubicin, paclitaxel, etoposide, cisplatin, bleomycin, mitomycin-C and topotecan, which resulted in enhanced generation of ROS, then cell death in human ovarian cancer cell lines; OVCAR-3, OVCAR-4, OVCAR-5, OVCAR-8, SKOV-3, and TYK-nu (Ozaki et al. 2008). This showed that increased generation of ROS play a role in HDAC-mediated cell death. It seems that HDACi-induced cell death is through extrinsic apoptotic pathway, which involves the cross-linking of certain death-inducing receptors like Fas by their legends (Drummond et al. 2005) and consequent activation of caspases 3, 8 and 9. In addition to this, HDACi utilises the intrinsic apoptotic pathway through the disruption of outer mitochondrial membrane integrity and later release of cytochrome c and other pro-apoptotic molecules. The involvement of these factors and pathways in the anti-cancer activity of HDACi need to be investigated. In addition, the effects of HDACi can be cell-type-dependent, and different HDACi can have different effects in the same cell type as shown by (Ozaki et al. 2008). This possibly relates to the HDAC-specificity profiles of the different compounds and HDACs profile in different cell lines since different HDAC isoforms contribute selectively to the development of ovarian cancer.

Another type of HDACi is sphingosine-1-phosphate (S1P). S1P is a lipid mediator that can act intracellularly as an HDAC inhibitor (Hait et al. 2009). S1P regulates histone acetylation by binding to HDAC1 and HDAC2. As S1P directly inhibits HDACs, it plays an essential role in epigenetic regulation of nuclear expression. S1P is aberrantly expressed in ovarian cancer and regulates key processes involved in ovarian cancer initiation and progression. Molecular agents that block S1P mediated signalling induce cellular apoptosis or inhibit ovarian cancer cell proliferation. Such evidence highlights a potential role for S1P as a drug target in ovarian cancer (Dai et al. 2014).

**Fig. 3:**
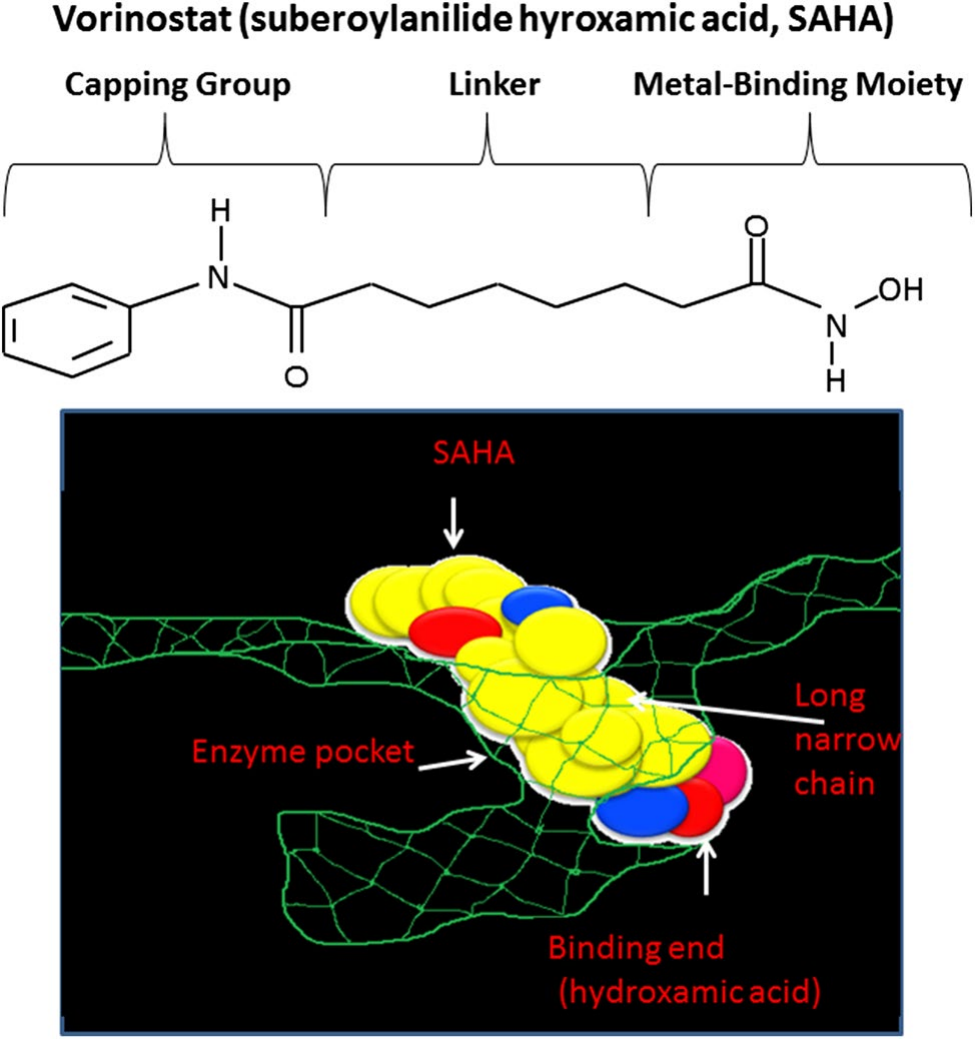
Vorinostat inhibits HDAC activity by binding to the pocket of the catalytic site. The hydroxamic acid moiety of vorinostat binds to a zinc atom (*pink*); this allows the rest of the molecule to lie along the surface of the HDLP protein. Adapted from Marks et al. (2001)

SAHA has been approved by the US Food and Drug Administration for the treatment of cutaneous T-cell lymphoma (CTCL), a form of non-Hodgkin’s lymphoma. Romidepsin is the second HDAC inhibitor to be approved by the FDA for the treatment of CTCL. Romidepsin has a very different chemical structure compared to vorinostat (Fig. 3). Romidepsin is a selective inhibitor for class I HDACs, whereas vorinostat is a pan-HDAC inhibitor (Campas-Moya 2009). Most HDACi are not specific to subclasses of HDAC enzymes. They inhibit many of the known mammalian HDACs. There has been increased attention over the past several years to create selective HDAC inhibitor. Given that specific HDAC isoform has specific function in cancer, selective HDAC inhibitors will be very useful in cancer treatment. This led to several class-selective and some isoform-selective inhibitors such as MS-275. MS-275 is more active against HDAC1 than against HDAC3 (Hu et al. 2003). Depsipeptide is more potent against HDAC1 and HDAC2 than against HDAC4 and HDAC6 (Furumai et al. 2002). 2f-MC1568 and 4a-MC1511 HDAC inhibitors are derivative of aroyl-pyrrolyl-hydroxyamides (APHAs) 2f-MC1568 was selective to class II but with poor inhibitory activity; however, 4a-MC1511 showed effective inhibitory activity against class II and to less extent to class I (Mai et al. 2005). Therefore, further studies are necessary in order to create HDAC isoform specific inhibitors.

The effect of HDACi in the treatment of epithelial ovarian cancer has been reported by Takai et al. (2006). The study investigated the effect of scriptaid, a member of the hydroxamic class of HDAC inhibitors that is selective for class I HDACs on Ishikawa endometrial cancer cell line, SKOV-3 ovarian cancer cell line, and normal human endometrial epithelial cells. The data showed that Scriptaid induced growth inhibition and increased acetylation of H3 and H4 histone for both endometrial and ovarian cancer cell lines. While normal endometrial epithelial cells were viable following treatment with similar doses of Scriptaid. The study showed the effectiveness of HDACi in mediating tumour cell selective killing compared with the normal cell. This may be because cancer cells have several genetic defects and, unlike normal cells, are unable to reverse the adverse effects of HDAC inhibitors. Vorinostat has been reported to induce cell death and increase caspase-3 activity in three ovarian cancer cell lines (OVCAR-3, SK-OV-3, and A2780) and in primary ovarian cancer cells isolated from malignant ascites collected from five patients with stage III ovarian carcinomas.

Furthermore, 2780AD, a doxorubicin-resistant ovarian cancer cell line cells were responsive to vorinostat (Sonnemann et al. 2006). Pre-treatment with vorinostat and trichostatin A (TSA) has been shown to enhance the cytotoxicity of DNA-targeting agents such as VP-16, ellipticine, doxorubicin, and cisplatin but not that of drugs which do not target the DNA, like the antimetabolite 5-fluorouracil (Kim et al. 2003). In addition, valproic acid (2-propylpentanoic acid; VPA), an HDAC inhibitor that inhibits both class I and II HDACs, has been reported to have synergistic cytotoxicity with cisplatin in human ovarian carcinoma cells (SK-OV-3, OVCAR-3, TOV-21G, A2780 and A2780/CP70) and can restore platinum sensitivity in the acquired cisplatin-resistance cells (Lin et al. 2008). Pre-clinical in vitro studies on the effect of PXD101, a class I and II HDAC inhibitor showed this HDACi exhibit single-agent antitumour activity on human A2780 ovarian cancer xenografts. This effect improved when combined with carboplatin treatment (Qian et al. 2006).

Clinical trials showed the limited therapeutic of some of HDACi such as TSA and trapoxin because they have poor bioavailability and toxic side effects at high doses. Other HDACi are degraded after i.v. administration like sodium butyrate and phenylbutyrate thus requiring high doses to achieve maximum tumour killing (Warrell et al. 1998). It has been shown that vorinostat-treated ovarian cancer cell lines and in primary cancer cells derived from malignant ascites obtained from five patients with stage III ovarian cancer had cytotoxic effect as a single agent (Sonnemann et al. 2006). A phase II trial of vorinostat in platinum-resistant/refractory epithelial ovarian patients showed progression-free survival over 6 months, with few having a partial response. However, vorinostat has toxic effects such as thrombocytopenia, neurologic complaints and pain which limit its effectiveness (Modesitt et al. 2008). This provides an evidence that HDAC inhibitors could have a potential role in the treatment of ovarian cancer, but there is need to develop HDAC inhibitor that has a tolerable side effect and efficient in destroying cancer cells.

## Conclusions and future perspectives

Ovarian cancer comprises a heterogenous group of tumours that are associated with a specific prognosis and drug resistance patterns. At present, there is a growing need for novel therapy to improve overall survival and disease-free survival hence reducing ovarian cancer related mortality. Addressing resistance to chemotherapy should be one of the strategies to target resistance to chemotherapy. As most drug resistance is at the level of gene expression, HDAC inhibitors are amongst the most promising therapeutic targets for cancer treatment due to their critical role in the regulation of transcription of key genes controlling important cellular functions such as cell proliferation, cell cycle regulation and apoptosis (Spiegel et al. 2012). While the preliminary results are promising, robust clinical trials are needed to ascertain whether this treatment offers beneficial clinical outcomes with tolerated side-effect profiles. A number of other drugs that target unique cancer pathways are undergoing trials. Given the complexity of disease management and the vast armamentarium of available drugs, utilising genetic tumour profiles has the potential to stratify patients and help select the best drug regimen that provides a superior benefit to risk ratio for each individual patient.
